# Effects of an Arbuscular Mycorrhizal Fungus on the Growth of and Cadmium Uptake in Maize Grown on Polluted Wasteland, Farmland and Slopeland Soils in a Lead-Zinc Mining Area

**DOI:** 10.3390/toxics10070359

**Published:** 2022-06-30

**Authors:** Jiaxin Chen, Jianfang Guo, Zuran Li, Xinran Liang, Yihong You, Mingrui Li, Yongmei He, Fangdong Zhan

**Affiliations:** 1 College of Resources and Environment, Yunnan Agricultural University, Kunming 650201, China; jiaxinchen96@126.com (J.C.); guojianfang2022@163.com (J.G.); lxr8900@live.com (X.L.); m15330490793@163.com (Y.Y.); limingrui@ynau.edu.cn (M.L.); heyongmei06@126.com (Y.H.); 2 College of Horticulture and Landscape, Yunnan Agricultural University, Kunming 650201, China; lizuran@ynau.edu.cn

**Keywords:** arbuscular mycorrhizal fungus, cadmium-polluted soil, root morphology, root exudates, cadmium uptake

## Abstract

Arbuscular mycorrhizal fungi (AMF) exist widely in soil polluted by heavy metals and have significant effects on plant growth and cadmium (Cd) uptake. Cd contents differ among wasteland, farmland and slopeland soils in a lead-zinc mining area in Yunnan Province, Southwest China. The effects of AMF on maize growth, root morphology, low-molecular-weight organic acid (LMWOA) concentrations and Cd uptake were investigated via a root-bag experiment. The results show that AMF increased maize growth on Cd-polluted soils, resulting in increases in root length, surface area, volume and branch number, with the effects being stronger in farmland than in wasteland and slopeland soils; increased malic acid and succinic acid secretion 1.3-fold and 1.1-fold, respectively, in roots on farmland soil; enhanced the iron- and manganese-oxidized Cd concentration by 22.6%, and decreased the organic-bound Cd concentration by 12.9% in the maize rhizosphere on farmland soil; and increased Cd uptake 12.5-fold and 1.7-fold in shoots and by 25.7% and 86.6% in roots grown on farmland and slopeland soils, respectively. Moreover, shoot Cd uptake presented significant positive correlations with root surface area and volume and LMWOA concentrations. Thus, these results indicated the possible mechanism that the increased maize Cd uptake induced by AMF was closely related to their effect on root morphology and LMWOA secretion, with the effects varying under different Cd pollution levels.

## 1. Introduction

With the continuous development of industry and agriculture, mismanaged mining and utilization of mining resources has led to different degrees of heavy metal pollution in the soil around mining areas [1]. According to the National Soil Pollution Survey Report published in 2014, the excess level of heavy metals in arable soil in China was 19.4%, wherein the excess level of cadmium (Cd) was 7%, ranking first among heavy metal pollutants [2]. Soil Cd pollution can reduce soil fertility, thereby inhibiting crop growth [3,4].

Arbuscular mycorrhizal fungi (AMF) are obligate symbiotic fungi that exist widely in nature and can form symbiotic systems with more than 80% of land plants [5,6]. Under Cd stress, AMF can form symbiotic relationships with most plants, promote plant growth [7] and affect the uptake of Cd by plants [8]. AMF have a large mycelial network, especially their extracellular mycelial network, which can promote the absorption and utilization of mineral nutrients and water by plants, enhance the root morphology of plants, cause changes in root exudates, change the availability of Cd in soil and promote plant growth [9]. During the process of heavy metal absorption by plants, low-molecular-weight organic acids (LMWOAs) in root exudates can promote complexation and chelation reactions, decrease the availability of heavy metals and reduce the toxicity of Cd to plants [10,11]. Consequently, AMF have the ecological function of promoting plant growth and enhancing plant Cd tolerance. However, research on the ecological function of AMF in soils with different Cd pollution levels is relatively limited.

The Huize lead-zinc mine, located in eastern Yunnan Province, is the most important lead-zinc mining, smelting and production base in China [12]. Long-term mining and smelting activities have led to the outward diffusion of a large number of heavy metals through dust and wastewater, contaminating the surrounding farmland and slopeland soil, in which Cd is the primary pollutant [13]. In particular, in the vicinity of smelters, soil Cd pollution is particularly severe and has caused some areas to become wastelands [14]. There are differences in soil physical and chemical properties and heavy metal pollution characteristics under different Cd pollution levels, which are related to the degree of influence of heavy metal contents in soil [15]. Different Cd concentrations have different effects on plant growth, especially under high Cd stress levels, and the effects are most obvious in plant tissue [16].

In this study, soils with different Cd pollution levels (wasteland, farmland and slopeland soils) were collected in the Huize lead-zinc mining area of Yunnan Province, Southwest China. Maize (*Zea mays* L.) was used as the host plant. A root-bag experiment was used to analyze the effects of AMF on maize growth, root morphology, exudate concentration, rhizosphere Cd morphology and plant Cd uptake. The effects of AMF on maize growth and Cd uptake in polluted soils with different Cd contents in a lead-zinc mining area were studied, gaining insight into the ecological functions of AMF in lead-zinc mining areas and providing a theoretical basis for the ecological restoration of heavy metal-contaminated farmland soil. We hypothesized that there would be an increase in Cd uptake in maize inoculated with AMF along with changes in root morphology and the secretion of LMWOAs, and that the effect would vary among polluted soils with different Cd contents.

## 2. Materials and Methods

### 2.1. Test Materials

Mountain red soils with different Cd levels, namely, wasteland soil (26°34′30″ N, 103°37′18′′ E), farmland soil (26°34′48″ N, 103°38′38″ E) and slopeland soil (26°37′40″ N, 103°41′54″ E), were collected in a lead-zinc mining area in Huize County, Yunnan Province, Southwest China. The soil physicochemical parameters are shown in Table 1. The naturally air-dried soils were sieved through a 2 mm mesh, autoclaved at 121 °C for 2 h and kept at room temperature for 3 d until use.

The total Cd content of wasteland, farmland and slopeland soils was 84.3 times, 22.3 times and 14.3 times the soil pollution risk screening value for agricultural land (0.3 mg/kg Cd), respectively (GB15618-2018) [17].

The tested maize (Huidan 4) is the main variety cultivated around the lead-zinc mining area in Huize County. Seeds of the same size were selected and disinfected with 75% ethanol (soaked for 1 min) and 10% sodium hypochlorite (soaked for 10 min), placed in sterilized petri dishes lined with soaked filter paper and incubated at a constant temperature of 25 °C for 3 d. After the seeds had germinated, infection-free seedlings with consistent growth were selected for use [18].

The test strain was *Funneliformis mosseae*, which was provided by the Institute of Plant Nutrition and Resources, Beijing Academy of Agriculture and Forestry Sciences (BGC YN05, 1511C0001BGCAM0013). AMF colonized the roots of the host plant, maize, to form a typical mycorrhizal system, which showed continuous expansion of the AMF fungal community and contained 25 to 28 spores per gram of soil after propagation.

### 2.2. Experimental Design

The root-bag experiment was carried out in the greenhouse of the eastern campus of Yunnan Agricultural University. The wasteland, farmland and slopeland soil samples were divided into control and treatment groups, with no inoculation as the control (CK) and inoculation with AMF as the treatment; each group had 4 replicates, with a total of 24 pots. The root bags were made of 400-mesh (38 μm) nylon with a diameter of 15 cm and a height of 20 cm. A total of 0.8 kg of sterilized soil was placed in each bag, with 100 g of inoculum spread on top under the germinating corn seeds. In the CK group, 100 g of inactivated fungicide was spread instead. We spread 0.2 kg of sterilized soil over the AMF inoculum and germinated maize seeds. The root bags were placed in medium pots (35 cm in diameter) with 2.5 kg of sterilized soil. During the experiment, natural light was applied, and we maintained the soil water concentration at 60–70% of the field water content. The indices were measured 60 d after planting.

### 2.3. Determination of the Arbuscular Mycorrhizal Fungal Colonization Rate and Spore Number

Maize roots were collected. Some tender roots were selected and cleaned with distilled water, cut into 1 cm-long root segments and placed in a 10 mL centrifuge tube, followed by the addition of 10% KOH solution and decolorization in a constant-temperature water bath (90 ℃) for 30 min until transparent. The roots were rinsed with sterile water 3 times, and 2% HCl was added to acidify the samples for 5 min. The samples were rinsed again with sterile water 3 times and stained with acid fuchsin staining solution for 3 h. After decolorization, a relatively complete root segment was selected and arranged on a slide under a coverslip. Mycelial infection was observed under an electron microscope [19], and the arbuscular mycorrhizal fungal infection rate was calculated by the cross method [20].

Spore number was determined by the wet sieving-decantation method. For this, 10 g of naturally dried rhizosphere soil was placed in a beaker, and 500 mL of sterile water was added, followed by clockwise stirring. Then, the sample was allowed to stand for 10 s and then sieved through an 80-mesh sieve followed by a 400-mesh sieve. The residue on the 400-mesh sieve was collected, transferred to a centrifuge tube and centrifuged at 3000 r/min for 5 min. The supernatant was collected, mixed with 50% sucrose solution and centrifuged for 10 min. The supernatant was filtered through a 0.45 µm membrane, and the spores of AMF were observed and counted under a microscope [21].

### 2.4. Extraction and Determination of the Root Exudates

The root exudates were collected by hydroponics [22]: Whole maize plants were collected, and the roots were cleaned three times with tap water and distilled water. The cleaned plants were transferred into 200 mL of 5 mg/L thymol solution for 3 min, then transferred into 200 mL of 0.005 mol/L calcium chloride solution collection bottles, wrapped with tin foil and cultured under dark conditions for 16 h. The collected solution was evaporated by rotation at 45 °C, condensed to 10 mL, filtered by 0.45 μm and frozen at −20 °C until testing.

LMWOAs were detected by high-performance liquid chromatography [23]. The detection conditions were as follows: The chromatographic column was an Agilent Zorbax SB-C18 (250 × 4.6 mm ID), the mobile phase was 10 mmol/L potassium dihydrogen phosphate solution (pH = 2.45), the column temperature was 35 °C, the flow rate was 1 mL/min, the injection volume was 10 μL, the detection wavelength was 214 nm, and the analysis time was 10 min.

### 2.5. Determination of Plant Height, Biomass and Root Morphology

Tape measures were used to measure plant height. The plants were divided into shoots and roots with tap water and then distilled water 2–3 times. The root system was scanned with a root scanner, and the scan image was saved. Root morphological parameters were analyzed using the RHIZO root analysis system. The maize samples were dried at 105 °C for 30 min, dried at 75 °C to a constant weight (which was recorded as the dry weight) and crushed through a 0.25 mm sieve for later use. Mycorrhizal dependence was calculated as follows [24]: mycorrhizal dependence (MD%) = inoculation treatment biomass (M^+^)/non-inoculation treatment biomass (M^−^) × 100%.

### 2.6. Determination of Cd Morphology in the Rhizosphere

The modified fractionation (Tessier) method was used to determine Cd speciation in soil [25]. One gram of air-dried soil that passed through a 2 mm pore size nylon sieve was weighed and placed in a 50 mL centrifuge tube. Then, 8 mL of 1 mol/L MgCl_2_ solution was added into the centrifuge tube followed by shaking extraction for 1 h. Next, 25 mL of 0.5 mol/L HONH_3_Cl solution (pH = 2) was added to the residue, followed by extraction at 96 ℃ for 6 h with agitation from time to time. Then, 10 mL of 30% H_2_O_2_ solution was added to the second-step residue, followed by shaking at room temperature for 1 h and digestion in a water bath (85 °C) for 1 h; 10 mL of 30% H_2_O_2_ was added again, followed by digestion to 1–2 mL in a water bath (85 °C); after cooling, 50 mL of 1.0 mol/L NH_4_OAc solution (pH = 2) was added, followed by shaking at room temperature for 16 h. After each step of extraction, the soil and the extract were centrifuged at 3000 rpm for 10 min to allow separation. The concentration of exchangeable Cd, iron- and manganese-oxidized Cd and organic-bound Cd in the separated extract could be measured sequentially.

### 2.7. Determination of Plant Cd Concentration and Uptake

Plant Cd concentration was determined by a HNO_3_-H_2_O_2_ digestion method: 0.5 g of sample powder was weighed into the digestion tank and mixed with 5 mL of HNO_3_ overnight. Then, 3 mL of 30% H_2_O_2_ was added, the inner lid was closed, the stainless steel jacket was tightened, and the sample was placed in a constant-temperature drying oven at 160 °C for 4 h, with the digestive juices making the sample completely transparent. We poured the digestive liquid into a 50 mL volumetric flask and washed the inner jar and inner cover 3 times. Distilled water was added to the mark, and after mixing, the Cd concentration of the plants was determined by flame atomic absorption spectrophotometry. Then, the biomass was multiplied by Cd concentration to determine the Cd uptake level in maize plants [26]. The detection limit of Cd in the instrument was 0.005 μg/mL, and the concentration of Cd in the standard reference soil (GBW70404) was 0.35 ± 0.06 mg/kg. Standard reference soil was used as a quality control during analytical determinations. Appropriate quality controls included the use of CdCl_2_ as a standard solution. Recovery percentages of 96–108% were obtained for Cd with AAS. 

### 2.8. Data Processing and Statistical Analysis

The test data were the average of 4 replicates. Microsoft Excel 2013 was used to process the data to calculate the average and standard deviation, expressed as the mean ± standard deviation. SPSS 23.0 was used to perform one-way analysis of variance (ANOVA) on the data, and the Duncan method was used to test the difference in the average value of each treatment at the 0.05 level. Origin Pro 9.0 was used for charting. Two-way ANOVA was performed on the data with AMF and soils with different Cd contents as factors, and Pearson correlation analysis was performed on the data for plant Cd uptake and maize root morphology and root exudates.

## 3. Results

### 3.1. Colonization of Arbuscular Mycorrhizal Fungi

As shown in Table 2, AMF in wasteland, farmland and slopeland soils successfully established a symbiotic relationship with maize roots. The AMF infection rates of maize roots were 30.8%, 36.8% and 40.4%, and the numbers of spores were 60, 64 and 57, respectively, demonstrating that the differences in the Cd contents of the soils affected the AMF infection rate and the number of spores.

### 3.2. Effects of Arbuscular Mycorrhizal Fungi on Maize Growth

As shown in Table 3, AMF on farmland and slopeland soils significantly increased the maize plant height 3.0-fold and by 97.7%, respectively; the shoot biomass increased 19.0-fold and 3.4-fold, respectively; the root biomass increased 4.7-fold and 2.3-fold, respectively; and the plant biomass increased 13.1-fold and 3.2-fold, respectively. The mycorrhizal dependence of maize on wasteland, farmland and slopeland soils was 110.4%, 1413.3% and 416.6%, respectively. The results show that AMF promoted maize growth on farmland soil better than on wasteland and slopeland soils under the root-bag experiment. Two-way ANOVA showed that AMF and soil type had extremely significant effects on maize plant height, root and shoot biomass, and mycorrhizal dependence. The interaction between the two affected maize plant height, root and shoot biomass, and mycorrhizal dependence.

### 3.3. Effects of Arbuscular Mycorrhizal Fungi on Maize Root Morphology

As shown in Table 4, AMF on both farmland and slopeland soils significantly increased maize root length, root surface area, root volume and branch number. On farmland soil, these parameters increased 4.4-fold, 6.6-fold, 6.8-fold and 5.1-fold, respectively, and on slopeland soil, these parameters increased by 37.2%, 40.4%, 39.7% and 43.1%, respectively, but AMF significantly reduced the average root diameter by 20.9% and 18.4% in farmland and slopeland soils, respectively. In conclusion, AMF on farmland soil improved maize root morphology better than AMF on wasteland and slopeland soils. Two-way ANOVA showed that AMF and soil type had extremely significant effects on root length, root surface area, root average diameter, root volume and branch number, and the interaction between AMF and soil type had extremely significant effects on root length, root surface area, root volume and branch number.

### 3.4. Effects of Arbuscular Mycorrhizal Fungi on the Secretion of Low-Molecular-Weight Organic Acids by Maize Roots

**Figure 1:**
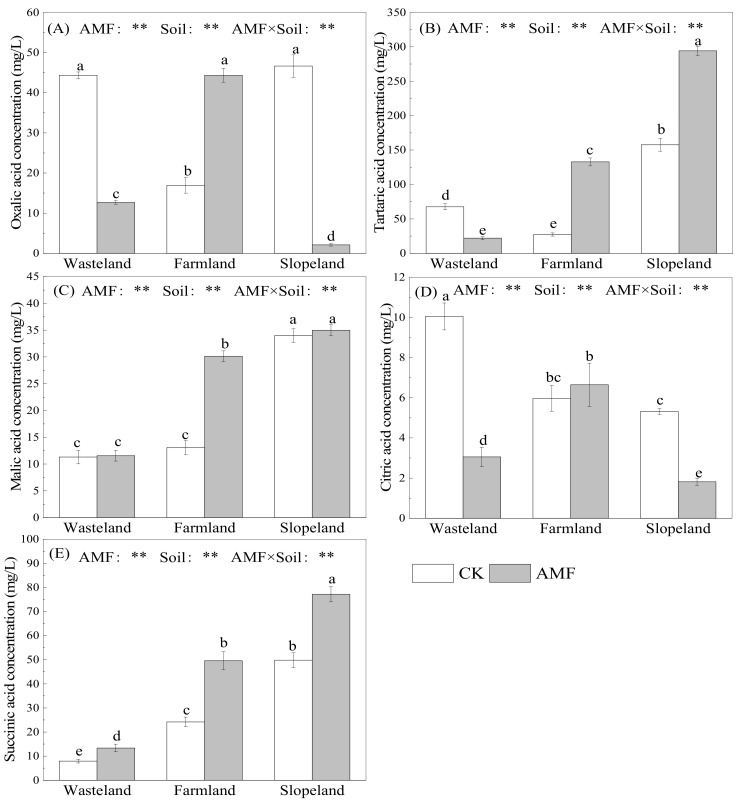
Effects of AMF on low-molecular-weight organic acid concentrations secreted by maize roots. (**A**): the concentration of oxalic acid secreted by maize roots with and without AMF inoculation; (**B**): the concentration of tartaric acid secreted by maize roots with and without AMF inoculation; (**C**): the concentration of malic acid secreted by maize roots with and without AMF inoculation; (**D**): the concentration of citric acid secreted by maize roots with and without AMF inoculation; (**E**): the concentration of succinic acid secreted by maize roots with and without AMF inoculation. The different lowercase letters indicate significant differences among treatments; “**” mean *p* < 0.01 according to two-way ANOVA.

AMF significantly reduced the concentrations of oxalic acid and citric acid secreted by maize roots in wasteland and slopeland soils. The concentration of oxalic acid secreted by maize roots decreased by 71.4% and 95.5%, and the concentration of citric acid secreted by maize roots decreased by 69.6% and 65.8%, in wasteland and slopeland soils, respectively. Nevertheless, AMF on farmland soil significantly increased the concentrations of oxalic acid and citric acid secreted by maize roots (1.6-fold and by 11.3%, respectively) (Figure 1A,D). AMF on farmland and slopeland soils significantly increased the concentration of tartaric acid secreted by maize roots (3.8-fold and by 86.5%, respectively); however, AMF on wasteland soil significantly decreased the concentration of tartaric acid secreted by maize roots, by 67.5% (Figure 1B). AMF on farmland soil increased the concentration of malic acid secreted by maize roots 1.3-fold, but had no significant effect in wasteland and slopeland soils (Figure 1C). In wasteland, farmland and slopeland soils, AMF significantly increased the concentration of succinic acid secreted by maize roots by 68.9%, 1.1-fold and by 54.8%, respectively (Figure 1E). In conclusion, AMF in the three soils with different Cd contents had different effects on the concentrations of LMWOAs secreted by the maize roots. Two-way ANOVA showed that AMF, soil type and their interaction had extremely significant effects on the concentrations of LMWOAs secreted by the maize roots.

### 3.5. Effects of Arbuscular Mycorrhizal Fungi on Cadmium Speciation and Availability in Soil

**Figure 2:**
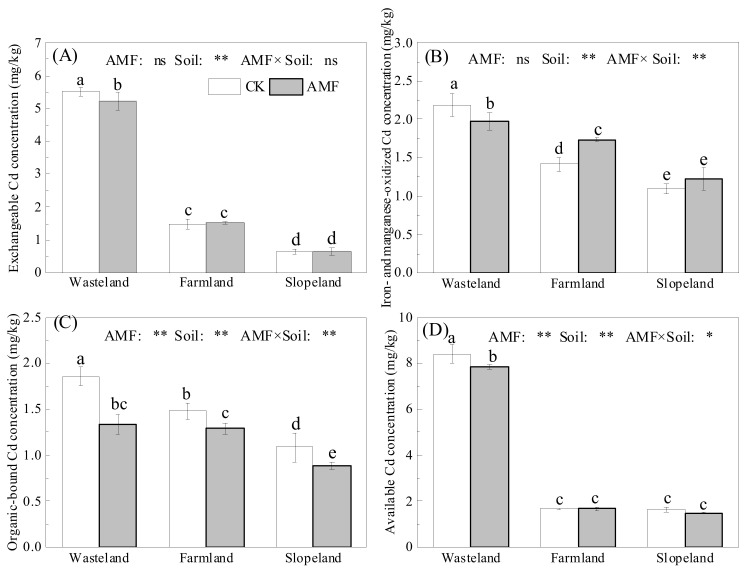
Effects of AMF on the morphology and availability of soil cadmium. (**A**): the concentrations of exchangeable Cd in with and without AMF inoculation soils; (**B**): the concentrations of iron- and manganese-oxidized Cd in with and without AMF inoculation soils; (**C**): the concentrations of organic-bound Cd in with and without AMF inoculation soils; (**D**): the concentrations of available Cd in with and without AMF inoculation soils. The different lowercase letters indicate significant differences among treatments; “ns”, “*” and “**” mean no significance, *p* < 0.05 and *p* < 0.01 according to two-way ANOVA, respectively.

AMF in wasteland soil significantly decreased the concentrations of exchangeable and available Cd, by 5.3% and 6.7%, respectively (Figure 2A,D). AMF increased the concentrations of iron- and manganese-oxidized Cd in farmland soil by 22.6% but significantly reduced the concentration by 9.4% in wasteland soil (Figure 2B). In wasteland, farmland and slopeland soils, AMF significantly decreased the organic-bound Cd concentration by 28.2%, 12.9% and 18.7%, respectively (Figure 2C). Thus, AMF in the three soils with different Cd contents had different effects on the morphology and availability of rhizospheric Cd. Two-way ANOVA showed that AMF had a significant effect on the soil organic binding state and the available Cd concentration. The soil type had significant effects on Cd speciation and availability. The interaction between the two had significant or extremely significant effects on the concentrations of iron- and manganese-oxidized Cd, organic-bound Cd and available Cd.

### 3.6. Effects of Arbuscular Mycorrhizal Fungi on Cadmium Concentration and Uptake in Maize

**Figure 3:**
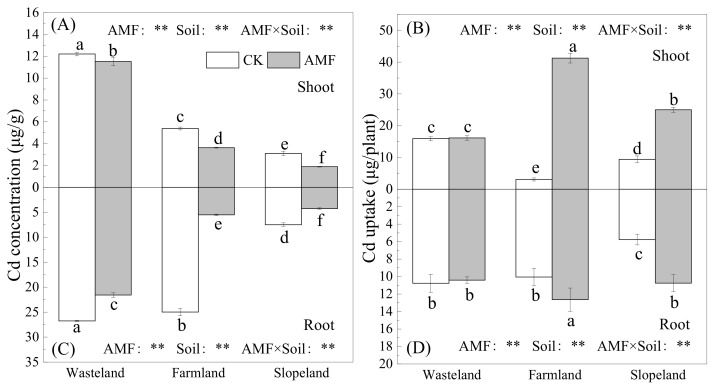
Effects of AMF on cadmium concentration and uptake in maize. (**A**): the shoot Cd concentrations of maize with and without AMF inoculation; (**B**): the shoot Cd uptake of maize with and without AMF inoculation; (**C**): the root Cd concentrations of maize with and without AMF inoculation; (**D**): the root Cd uptake of maize with and without AMF inoculation. The different lowercase letters indicate significant differences among treatments; “**” mean *p* < 0.01 according to two-way ANOVA.

As shown in Figure 3, AMF in wasteland, farmland and slopeland soils significantly reduced the shoot and root Cd concentrations of maize, wherein the shoot Cd concentration decreased by 5.6%, 32.6% and 39.2%, respectively, and the root Cd concentration decreased by 19.4%, 77.9% and 43.1%, respectively (Figure 3A,C). In farmland and slopeland soils, AMF significantly increased Cd uptake in maize shoots and roots, with Cd uptake in the shoots increasing 12.5-fold and 1.7-fold, and Cd uptake in roots increasing by 25.7% and 86.6% in farmland and slopeland soils, respectively (Figure 3B,D). Hence, in the root-bag experiment, AMF in the three soils with different Cd contents had different promoting effects on the concentration and uptake of Cd in maize. Two-way ANOVA showed that AMF and soil type had significant effects on Cd concentration and Cd uptake in maize shoots and roots and that interactions of the two had effects on Cd concentration and uptake in maize shoots and roots.

### 3.7. Correlation Analysis

Correlation analysis showed that Cd uptake in maize shoots was significantly positively correlated with the root surface area (r = 0.575, *p* < 0.01) and root volume (r = 0.663, *p* < 0.01), with the concentrations of root tartaric acid (r = 0.418, *p* < 0.05), malic acid (r = 0.448, *p* < 0.05) and succinic acid (r = 0.428, *p* < 0.05) secreted by the roots being positively correlated. Thus, the increase in Cd uptake in maize caused by AMF is closely related to improvement of root morphology and LMWOA secretion.

## 4. Discussion

The mycorrhizal infection rate is an important indicator of the affinity between mycorrhizal fungi and host plants [27]. Studies have shown that soil heavy metal contents can affect AMF infection in maize roots, which may be because high heavy metal contents inhibit the diffusion of AMF mycelia and spore germination [28]. In this study, AMF on wasteland, farmland and slopeland soils could effectively infect maize root systems, among which the effect of maize root infection was lowest in wasteland soil, followed by farmland and slopeland soils. However, mycorrhizal infection was still detected in maize roots on wasteland soil, which may be due to the strong tolerance of *F. mosseae* to Cd, which has a certain application value in the remediation of Cd-polluted soil.

Mycorrhizal dependence is the degree to which plants rely on mycorrhizae for maximum growth or yield after AMF colonize plant roots under certain soil fertility conditions [29]. Mycorrhizal effects depend not only on specific plant–fungal associations but also on specific environmental conditions [30]. The dependence of plants on mycorrhizae differed under the stress caused by different heavy metal concentrations [31]. In this study, the degree of dependence of maize on mycorrhizae differed among wasteland, farmland and slopeland soils, with farmland soil showing the highest value and wasteland soil showing the lowest value. This indicates that too high or too low a content of heavy metals in the soil may affect the dependence of maize on mycorrhizae.

The root system is an important organ for plants, absorbing water and nutrients from soil, and the growth status of the root system directly affects the growth and development of the plant [32]. Studies have shown that high contents of heavy metals in soil significantly inhibit plant growth, especially root growth, leading to changes in root morphology and physiology, thereby affecting the absorption of water and mineral nutrients by the root system [33]. Plants can adapt to heavy metal stress by changing the root morphology and distribution, affecting the absorption and accumulation of heavy metals in the plants [34]. In this study, it was found that AMF in farmland and slopeland soils significantly increased the root biomass, root length, root surface area, root volume and branch number of maize but decreased the average root diameter, which is consistent with the results of Yu et al. [35]. Studies have shown that the ability of plant roots to absorb water and nutrients mainly depends on the root surface area. In particular, fine roots (root diameter ≤2 mm) are the active part of plants that absorb nutrients because of their large specific surface area and large contact area with the soil [36].

The effects of AMF on plant growth differed among soils with different pollution levels [37,38]. In this study, the root-bag experiment showed that the root bag limited the growth space of maize roots, restricting the growth of the maize root system and resulting in the biomass of the inoculated treatment group on farmland and slopeland soils being much greater than that in the uninoculated treatment group. Therefore, the hyphae that grew out of the root bag in this case showed a substantial effect. AMF form a large mycelial network, and the extension of extracapsular-root mycelia enables plants to absorb additional mineral nutrients from areas that the roots cannot reach [39,40]. Therefore, AMF promote the absorption of mineral nutrients and water in plants [41], which is beneficial for adversity resistance and crop growth promotion [42]. In this study, the biomass in uninoculated wasteland soil was greater than that in uninoculated farmland soil, indicating that the growth of the plants was affected by not only the degree of Cd pollution but also other complex factors, such as soil nutrients.

In this study, the concentration of available phosphorus and potassium in maize rhizosphere soil decreased significantly after inoculation with AMF in farmland soil, and there was no significant difference between the wasteland and slopeland soils (Figure S1). In addition, the changes in the nitrogen, phosphorus and potassium concentrations in the shoots of the plants were inconsistent after AMF inoculation in the three soils (Figure S2), which may be related to the physical and chemical properties of the tested soils and the degree of heavy metal pollution in the soils. Among these changes, AMF inoculation in wasteland and farmland soils led to no significant difference in the shoot nitrogen and phosphorus concentrations, which may be attributed to the high concentration of Cd in the soil and the high stress on the maize plants, indicating that Cd stress was the dominant factor.

Under heavy metal stress, plants can resist the toxicity of heavy metals through the secretion of LMWOAs from their roots [43]. Studies have shown that the LMWOAs secreted by roots can chelate with Cd in soil to form a “Cd–LMWOA” complex, thus inhibiting Cd uptake by plants [44]. This study found that AMF affected the secretion of oxalic acid, tartaric acid, malic acid, citric acid and succinic acid in roots, but the changes in LMWOA secretion from maize roots in wasteland, farmland and slopeland soils were not consistent. This indicates that in addition to the influence of the Cd stress level, soil physicochemical properties and soil nutrient status also affected the secretion of LMWOAs in roots. In a restricted growth space, excess root hyphae can expand the area available for heavy metal absorption, and the mycelia can better absorb heavy metals, fix heavy metals and reduce plant absorption of heavy metals [45]. Heavy metals enter mycelial cells, and specific heavy metal complex proteins and organic acids in the cells can combine with heavy metals to form heavy metal crystals or precipitates [46]. Some studies have found that AMF change factors such as rhizosphere soil pH by changing the secretion of LMWOAs in roots, indirectly changing soil Cd availability [47] and reducing the transfer of Cd from soil to plants [48].

Many factors affect the uptake of heavy metals in plants by AMF, such as the degree of heavy metal pollution and the types of AMF in different host plants and rhizosphere soil, affecting the accumulation of heavy metals in plants [49]. The sensitivity of AMF to different heavy metal pollutants is different, resulting in differences in the effects of AMF on the uptake of heavy metals in plants [50]. This study has shown that AMF in farmland and slopeland soils significantly reduced the Cd concentration in maize and increased Cd uptake in maize, but AMF had no significant effect on Cd uptake in maize from wasteland soil. These results indicate that AMF could have an ecological function and decrease the toxic effect of Cd on plants, but only under a certain concentration of heavy metal stress. AMF reduced the plant Cd concentration but increased maize Cd uptake. This is mainly because AMF significantly increase the biomass of maize under a certain level of heavy metal stress.

Overall, in the root-bag experiment in this study, although the root bag limited the space for the growth of maize roots, it facilitated the functions of the extracellular mycelia of AMF via a concentration effect, concentrating the LMWOAs secreted by the root system, promoting complexation and chelation with Cd, increasing Cd uptake and thus reducing the toxicity of Cd to plants. In this study, AMF had different effects on maize growth, root morphology, root exudates and plant Cd concentration and uptake in soils with three different Cd contents, which may be due to the differences in heavy metal pollution degree, nutrient concentration and other physical and chemical properties of soils with different Cd contents. Thus, the mechanism of action of AMF in soils with different Cd contents needs further study.

## 5. Conclusions

In the root-bag experiment conducted in this study, AMF improved maize growth, root morphology and LMWOA secretion and increased maize Cd uptake in three soils with different Cd contents. However, the effects of AMF varied among the three soils with different Cd concentrations. While maize growth was consistently promoted, Cd uptake was higher in farmland soil than in wasteland and slopeland soils. In addition, Cd uptake by maize shoots was significantly positively correlated with root morphology (root surface area, root volume) and the concentrations of LMWOAs secreted by roots. These results indicate that AMF regulated maize root morphology and LMWOA secretion, thereby promoting Cd uptake by maize. It is also noted that AMF assist in overall plant growth by seeking more nutrients, thereby affecting Cd uptake.

## Figures and Tables

**Table 1:** Soil physicochemical parameters.

Soils	pH	CEC (cmol/kg)	Total N (g/kg)	Total P (g/kg)	Available N (mg/kg)	Available P (mg/kg)	Available K (mg/kg)	Organic Matter (mg/kg)	Cd Content (mg/kg)
Wasteland	6.2	9.0	0.66	0.43	103.5	8.8	85.2	34.0	25.3
Farmland	6.4	9.3	1.64	0.47	86.3	13.3	122.5	22.3	6.7
Slopeland	6.8	26.0	0.44	0.49	34.5	22.8	63.6	3.1	4.3

**Table 2:** AMF infection characteristics. The data in the table are the mean ± standard deviation of 4 replicates, and the different lowercase letters indicate significant differences among treatments (*p* < 0.05), respectively.

Soils	Colonization Rate (%)	Spore Number (n/g)
Wasteland	30.8 ± 0.3 b	60 ± 1.2 b
Farmland	36.8 ± 0.3 a	64 ± 1.3 a
Slopeland	40.4 ± 0.6 a	57 ± 1.0 b

**Table 3:** Effects of AMF on maize plant height and biomass. The different lowercase letters indicate significant differences among treatments; “ns” “*” and “**” mean *p* < 0.05 and *p* < 0.01 according to two-way analysis of variance, respectively.

Soils	Treatment	Height (cm)	Shoot Biomass (g/Plant)	Root Biomass (g/Plant)	Plant Biomass (g/Plant)	Mycorrhizal Dependency (%)
Wasteland	CK	20.3 ± 0.85 d	1.30 ± 0.05 d	0.40 ± 0.04 c	1.70 ± 0.04 d	-
	AMF	20.9 ± 0.47 d	1.40 ± 0.04 d	0.48 ± 0.01 c	1.88 ± 0.05 d	110.4 ± 0.02 c
Farmland	CK	13.1 ± 0.91 e	0.57 ± 0.08 e	0.40 ± 0.04 c	0.97 ± 0.08 e	-
	AMF	52.6 ± 1.58 b	11.35 ± 0.38 b	2.29 ± 0.19 b	13.64 ± 0.38 b	1413.3 ± 1.29 a
Slopeland	CK	27.7 ± 0.70 c	3.03 ± 0.21 c	0.77 ± 0.07 bc	3.80 ± 0.23 c	-
	AMF	54.7 ± 1.16 a	13.25 ± 0.34 a	2.53 ± 0.21 a	15.78 ± 0.38 a	431.1 ± 0.25 b
Two-way analysis of variance						
AMF		**	**	**	**	**
Soil		**	**	**	**	**
AMF × Soil		**	**	*	**	*

**Table 4:** Effects of AMF on the morphology of maize roots. The different lowercase letters indicate significant differences among treatments; “ns” and “**” mean no significance and *p* < 0.01 according to two-way analysis of variance, respectively.

Soils	Treatment	Root Length (m)	Root Surface Area (cm^2^)	Average Root Diameter (mm)	Root Volume (cm^3^)	Branch Number (10^3^)
Wasteland	CK	7.1 ± 1.2 d	146.6 ± 12.9 d	0.66 ± 0.03 a	2.5 ± 0.1 e	6.6 ± 0.12 d
	AMF	12.2 ± 0.6 d	159.0 ± 14.9 d	0.56 ± 0.04 b	3.3 ± 0.4 d	7.2 ± 0.39 d
Farmland	CK	10.5 ± 0.9 d	95.7 ± 4.2 e	0.47 ± 0.02 c	1.1 ± 0.1 f	11.7 ± 2.16 d
	AMF	56.79 ± 8.5 c	727.5 ± 9.3 b	0.37 ± 0.04 de	8.6 ± 0.5 b	71.5 ± 1.6 c
Slopeland	CK	75.7 ± 4.9 b	687.9 ± 37.1 c	0.41 ± 0.05 d	7.4 ± 0.1 c	151.3 ± 9.8 b
	AMF	103.9 ± 5.0 a	965.9 ± 47.8 a	0.33 ± 0.04 e	10.3 ± 0.5 a	216.4 ± 1.3 a
Two-way analysis of variance						
AMF		**	**	**	**	**
Soil		**	**	**	**	**
AMF × Soil		**	**	ns	**	**

## Data Availability

Not applicable.

## Supplementary Materials

The following supporting information can be downloaded at: https://www.mdpi.com/article/10.3390/toxics10070359/s1, Figure S1: Effects of AMF on the concentrations of available nutrients in soil. Figure S2: Effects of AMF on the concentrations of nitrogen, phosphorus and potassium in the shoots of maize. Figure S3: Growth of control and AMF-inoculated maize on wasteland, farmland, and slopeland soils.
