# Supplementary material for: Effects of an Arbuscular Mycorrhizal Fungus on the Growth of and Cadmium Uptake in Maize Grown on Polluted Wasteland, Farmland and Slopeland Soils in a Lead-Zinc Mining Area
Source: Toxics. 2022 Jun 30;10(7):359. doi: 10.3390/toxics10070359 (PMC9322003; doi:10.3390/toxics10070359)
Supplement: Supplementary file 1 [file toxics-10-00359-s001.zip › toxics-1795930-supplementary.pdf]

toxics

Supplementary Materials: Effects of AMF on the growth of and cadmium uptake in maize grown on polluted wasteland, farmland and slopeland soils in a lead-zinc mining area

Jiaxin Chen[1], Jianfang Guo[1], Zuran Li[2], Xinran Liang[1], Yihong You[1], Mingrui Li[1], Yongmei He[1] and Fangdong Zhan[1]*

[1] College of Resources and Environment, Yunnan Agricultural University, Kunming 650201, Yunnan, China
[2] College of Horticulture and Landscape, Yunnan Agricultural University, Kunming 650201, China
* Correspondence: zfd97@ynau.edu.cn

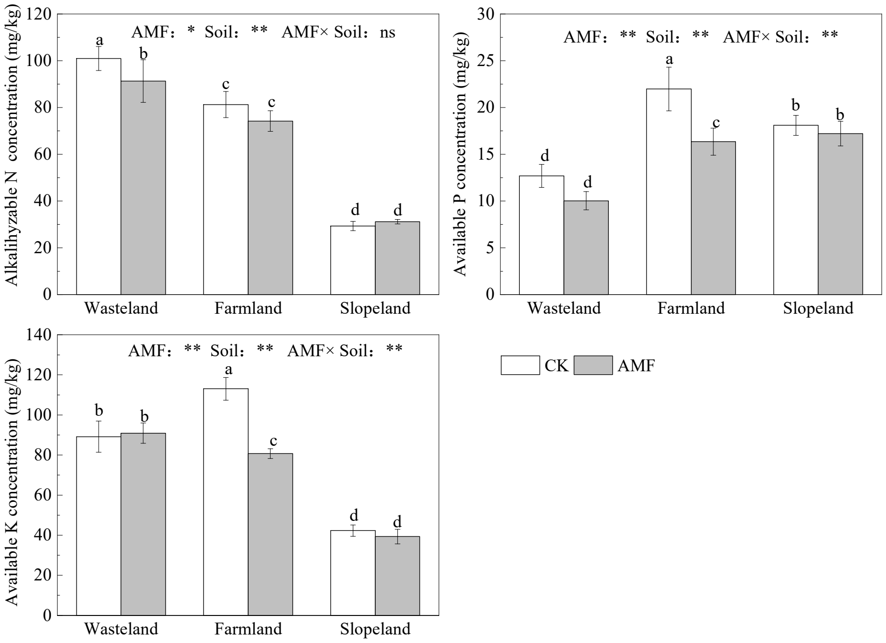

Figure S1. Effects of AMF on the concentrations of available nutrients in soil. The different lower-case letters indicate significant differences among treatments; "*", and "**" mean p < 0.05 and p < 0.01 according to two-way ANOVA, respectively.

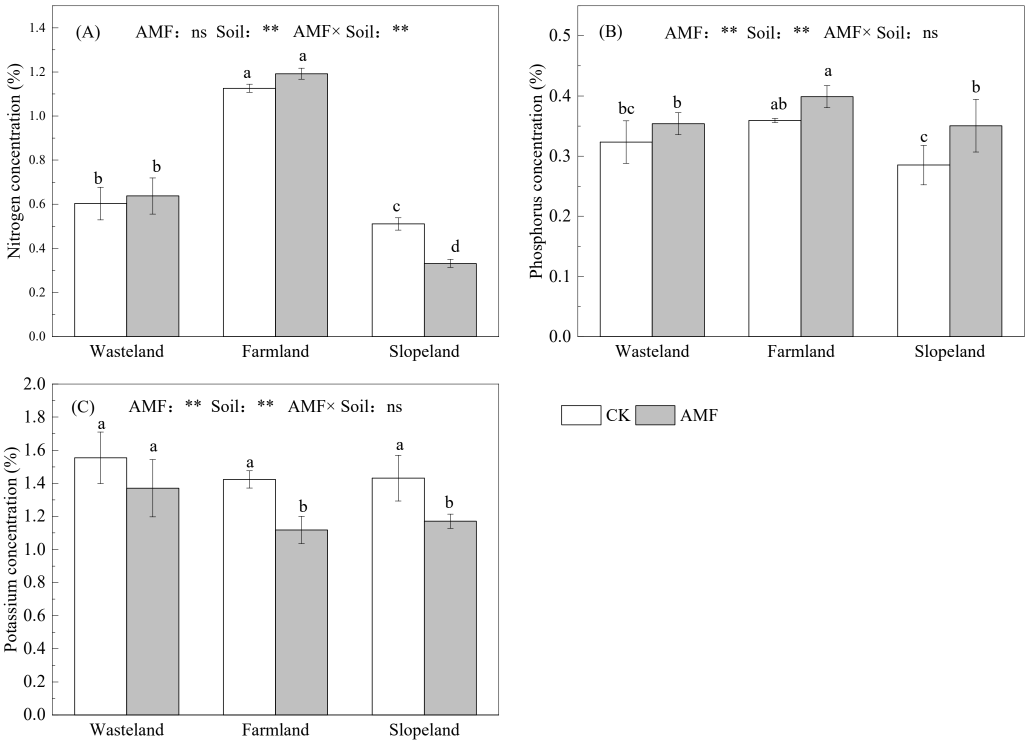

Figure S2. Effects of AMF on the concentrations of nitrogen, phosphorus and potassium in the shoots of maize. The different lowercase letters indicate significant differences among treatments; "*", and "**" mean $p < 0.05$ and $p < 0.01$ according to two-way ANOVA, respectively.

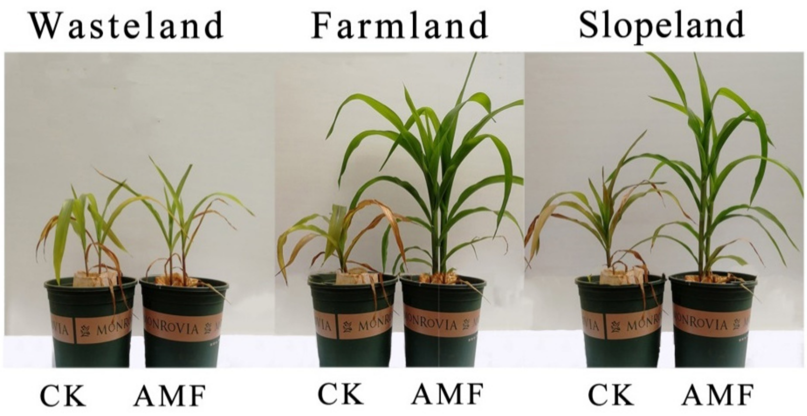

Figure S3. Growth of control and AMF-inoculated maize on wasteland, farmland, and slopeland soils.
